# Web-based pulmonary telehabilitation: a systematic review

**DOI:** 10.1038/s41533-024-00396-5

**Published:** 2024-11-16

**Authors:** Manuel Ayala-Chauvin, Fernando A. Chicaiza, Patricia Acosta-Vargas, Janio Jadan, Verónica Maldonado-Garcés, Esteban Ortiz-Prado, Gloria Acosta-Vargas, Mayra Carrión-Toro, Marco Santórum, Mario Gonzalez-Rodriguez, Camila Madera, Wilmer Esparza

**Affiliations:** 1 Centro de Investigación en Ciencias Humanas y de la Educación—CICHE, Facultad de Ingenierías, Ingeniería Industrial, Universidad Tecnológica Indoamérica, Ambato, Tungurahua Ecuador; 2 Facultad de Ingeniería y Ciencias Aplicadas, Universidad de las Américas, Quito, Pichincha Ecuador; 3 Centro de Investigación en Mecatrónica y Sistemas Interactivos—MIST, Facultad de Ingenierías, Ingeniería Industrial, Universidad Tecnológica Indoamérica, Ambato, Tungurahua Ecuador; 4 Facultad de Psicología, Pontificia Universidad Católica del Ecuador, Quito, Pichincha Ecuador; 5 Facultad de Ciencias de la Salud, Universidad de las Américas, Quito, Pichincha Ecuador; 6 Facultad de Medicina, Pontificia Universidad Católica del Ecuador, Quito, Pichincha Ecuador; 7 Facultad de Ingeniería de Sistemas, Escuela Politécnica Nacional, Quito, Pichincha Ecuador; 8 Facultad de Enfermería, Pontificia Universidad Católica del Ecuador, Quito, Pichincha Ecuador

**Keywords:** Rehabilitation, Quality of life

## Abstract

Web-based pulmonary telerehabilitation (WBPTR) can serve as a valuable tool when access to conventional care is limited. This review assesses a series of studies that explore pulmonary telerehabilitation programmes delivered via web-based platforms. The studies involved participants with moderate to severe chronic obstructive pulmonary disease (COPD). Of the 3190 participants, 1697 engaged in WBPTR platforms, while the remaining 1493 comprised the control groups. Sixteen studies were included in the meta-analysis. Web-based pulmonary telerehabilitation led to an increase in daily step count (MD 446.66, 95% CI 96.47 to 796.86), though this did not meet the minimum clinically important difference. Additionally, WBPTR did not yield significant improvements in the six-minute walking test (MD 5.01, 95% CI − 5.19 to 15.21), health-related quality of life as measured by the St. George’s Respiratory Questionnaire (MD − 0.15, 95% CI − 2.24 to 1.95), or the Chronic Respiratory Disease Questionnaire (MD 0.17, 95% CI − 0.13 to 0.46). Moreover, there was no significant improvement in dyspnoea-related health status, as assessed by the Chronic Respiratory Disease Questionnaire (MD − 0.01, 95% CI − 0.29 to 0.27) or the modified Medical Research Council Dyspnoea Scale (MD − 0.14, 95% CI − 0.43 to 0.14). Based on these findings, this review concludes that WBPTR does not offer substantial advantages over traditional care. While slight improvements in exercise performance were observed, no meaningful enhancements were noted in dyspnoea or quality of life metrics. Overall, WBPTR remains a complementary and accessible option for managing and monitoring COPD patients. However, further research and innovation are required to improve its efficacy and adapt it to various clinical environments.

## Introduction

Respiratory diseases are leading causes of death worldwide. In particular, chronic obstructive pulmonary disease (COPD) is one of the four most common causes of death, according to the World Health Organization^1^. Patients with COPD often suffer from physical inactivity due to fatigue, cough, dyspnoea, and recurrent chest infections. Such events can worsen the health condition of sufferers, affecting not only the organs directly related to COPD but also other systems of the human body ^2^. Evidence shows that to reduce the impact of long-term sequelae, pulmonary rehabilitation (PR) counteracts the effects of COPD by maximising the functional capacity of affected individuals and significantly improving their quality of life. Encouraging results are observed when PR is initiated early and maintained over a long continuous period^3^.

PR is a programme that includes several components: personalised physical exercise therapies for each patient (where specialists design programmes, assess progress, validate results, and so on), information to understand the disease (recognising that self-education plays a fundamental role in lifestyle modification), and professional interventions (to manage the emotional and psychological state of each patient)^4,5^. Especially in patients who have recovered from respiratory disease in intensive care units, the prolonged days of mechanical ventilation, dehydration, and fear of death can lead to stress, anxiety, and depression, necessitating psychological support in addition to PR^6^. Likewise, the social stigma associated with the disease also impacts both patients and their immediate family circle. Therefore, respiratory rehabilitation should focus not only on the organs affected by the disease (especially the lungs) but also on the side effects, such as the mental health of patients. In this context, social support for motivation, appropriate education, and feedback to understand the disease, approximate recovery times, and continuous progress monitoring can greatly enhance ongoing rehabilitation^7^.

In addition to appropriate care from medical staff, personal aspects such as health behaviour, self-determination, and commitment to recovery are directly related to each patient’s progress. However, not all users of the rehabilitation system show a positive interest in their recovery, and the education of each individual directly impacts this matter^8^. Understanding the causes, the recovery process, and the risks of deterioration allows patients to recognise the pathophysiology of the disease^9^. When professionally validated information is delivered to patients, the possibility of recovery increases in people with self-learning abilities^10^.

Although several studies demonstrate the efficacy of PR, it may be underutilised^11^. This is due to poor accessibility of rehabilitation centres, cost considerations, lack of awareness of their importance^12^, among other factors. Additionally, restrictions on mobility, saturation of healthcare systems, and social distancing resulting from the health emergency that began in China in December 2019 have exacerbated traditional pulmonary care^13^. These factors have spurred studies into various forms of pulmonary telerehabilitation, many leveraging ubiquitous technological tools, supported by the Internet.

Through conducting a systematic review of the current state of the art, this study aims to investigate web-based platforms for pulmonary telerehabilitation and evaluate their effectiveness using objective (quantitative assessment of different aspects of a patient’s lung function and physical capacity) and subjective (based on the patient’s perception and personal experience) data, particularly from trial-type studies. Given the extensive research on pulmonary telerehabilitation strategies, this review focuses exclusively on studies examining web-based platforms that offer methods for delivering rehabilitation. This analysis will provide insights into patient acceptance of such platforms and the identification of strengths and weaknesses inherent in these systems. Consequently, this review seeks to address how effective web-based pulmonary telerehabilitation (WBPTR) is compared to traditional care.

## Methods

This report adheres to the guidelines of the Preferred Reporting Items for Systematic Reviews and Meta-Analyses (PRISMA)^14^, and the protocol for extracting information was designed according to the Cochrane Handbook for Systematic Reviews of Interventions^15^.

### Why is it important to do this review?

As previously defined, a health emergency like the one witnessed in 2019 complicates aspects related to traditional care given by the medical centres. In this scenario, an online platform with feedback would enable rehabilitation sessions without exposing participants.

This study aims to identify accessible platforms that patients can use to sustain their rehabilitation sessions, highlighting the advantages and limitations of each. Additionally, the findings from this study will contribute to developing innovative approaches to address the current limitations identified in existing platforms.

### Information sources and search strategy

The search for articles encompassed several databases, including PubMed, Cochrane, Scopus, Science Direct, Google Scholar, and MEDLINE. Medical Subject Headings (MeSh) were employed to identify articles of interest. Depending on the search engine, strategies were formulated while maintaining a structure consisting of two parts, (i) Basic search requirements: Pulmonary rehabilitation, COPD, Chronic obstructive pulmonary disease, Web-based rehabilitation, Internet-based rehabilitation, Rehabilitation exercise, Pulmonary exercise, Respiratory function exercise; and (ii) Specific-oriented search: Self-management, Self-education, Virtual education, Rehabilitation, Therapy, Education. All these terms were searched using selective Boolean operators -AND- and -OR-.

### Selection criteria

Studies that met the criteria shown in Table 1 were eligible, regardless of whether they were abstracts or full texts. The studies (reported in either English or Spanish) were considered eligible only if the programmes incorporated web-based tools for delivering rehabilitation. The programmes must cover either extensive-type training (endurance exercises), intensive-type training (strengthening), or a combination of both. Breathing exercises or yoga were considered ineligible.

**Table 1:** Selection criteria considering participants, intervention, comparison and outcomes.

Participants:
• People aged 55 years and older
• With Severe and Moderate COPD
Intervention:
• Delivered using web-based platforms
• Programme with at least 4 weeks
• Session including intensive and extensive training
Comparison:
• WBPTR vs traditional care
Outcomes:
Main:
• Related to Exercise performance
• Related to quality of life
• Related to dyspnoea
Supplementary:
• Health status
• Measures related to fatigue and reduced activity
• Self-efficacy using questionnaires
• Exacerbation, hospitalisations

Therefore, three authors (M.A.C., F.A.C., P.A.V.) independently searched the databases. Subsequently, three authors (G.A.V., M.C.T., and M.S.) assisted in selecting the articles that met all the criteria for inclusion. Any disagreements in the selection were resolved through discussion and interpretation by two authors (F.A.C. and M.A.C.).

### Data extraction

Two authors (M.G.R., C.M.) independently extracted and reviewed information from eligible articles. Any disagreements were resolved through discussion, with interpretation by a third author (J.J.). In cases where information was not directly included in the full article, the authors referred to online appendices. When these were unavailable, missing information was requested from the original authors. To evaluate the performance of each study, the change in mean values from baseline to end of treatment was tabulated, standardising measures when different units were used (following the international system of units^16^). Additionally, the data were categorised for analysis and to derive conclusions, using forest plots for comparison.

### Data analysis

Authors standardised parameters as needed for data that could be processed through meta-analysis. The clinical utility of the assessed treatment effects was evaluated based on their corresponding minimum clinically important difference (MCID)^17^, whenever provided: 25.0 [20.0, 60.0] metres for the six-minute walking test (6MWT), 65 seconds for the endurance shuttle walk test (ESWT), 600 steps for daily step count (SPD), −4 for the St. George’s Respiratory Questionnaire (SGRQ), 0.5 for the Chronic Respiratory Disease Questionnaire and its dyspnoea sub-score (CRQ), −0.6 for the Clinical COPD Questionnaire score (CCQ), −2.5 for the COPD Assessment Test (CAT), and 1 point for the modified Medical Research Council dyspnoea Scale (mMRC).

The amount of heterogeneity (i.e., tau^2^) was estimated using the DerSimonian-Laird estimator^18^. In addition to the estimate of tau^2^, the Q-test for heterogeneity and the I^2^ statistic were reported^19^. Considering the heterogeneity in methodologies and variations in patient characteristics such as age, ethnicity, and economic conditions, a random-effects (RE) model was selected, in which I^2^ > 40 determines significant heterogeneity^20^. This choice allows flexibility in including future articles that have not been published or whose data have not yet been reported. All calculations were conducted using Jamovi v2.3 software to support the analysis^21^.

Studies where the information was challenging to ascertain or not quantifiable reported were tabulated separately. Additionally, if the data were included but did not meet the minimum number of studies required for meta-analysis (≥3), they were analysed one by one.

### Risk of bias assessment

Based on the Cochrane Handbook, two authors from a pool of three (J.J., V.M.G., M.S.) independently assessed the eligible studies for risk of bias using the following criteria: (a) selection bias: random sequence generation and allocation concealment; (b) performance bias: blinding of participants and personnel; (c) detection bias: blinding of outcome assessment; (d) attrition bias: incomplete outcome data; (e) reporting bias: selective reporting; and (f) other bias^15^. In cases where there was significant disagreement between the authors, a fourth author (F.A.C.) adjudicated the disagreement.

## Results

### Characteristics of the included studies

**Fig. 1:**
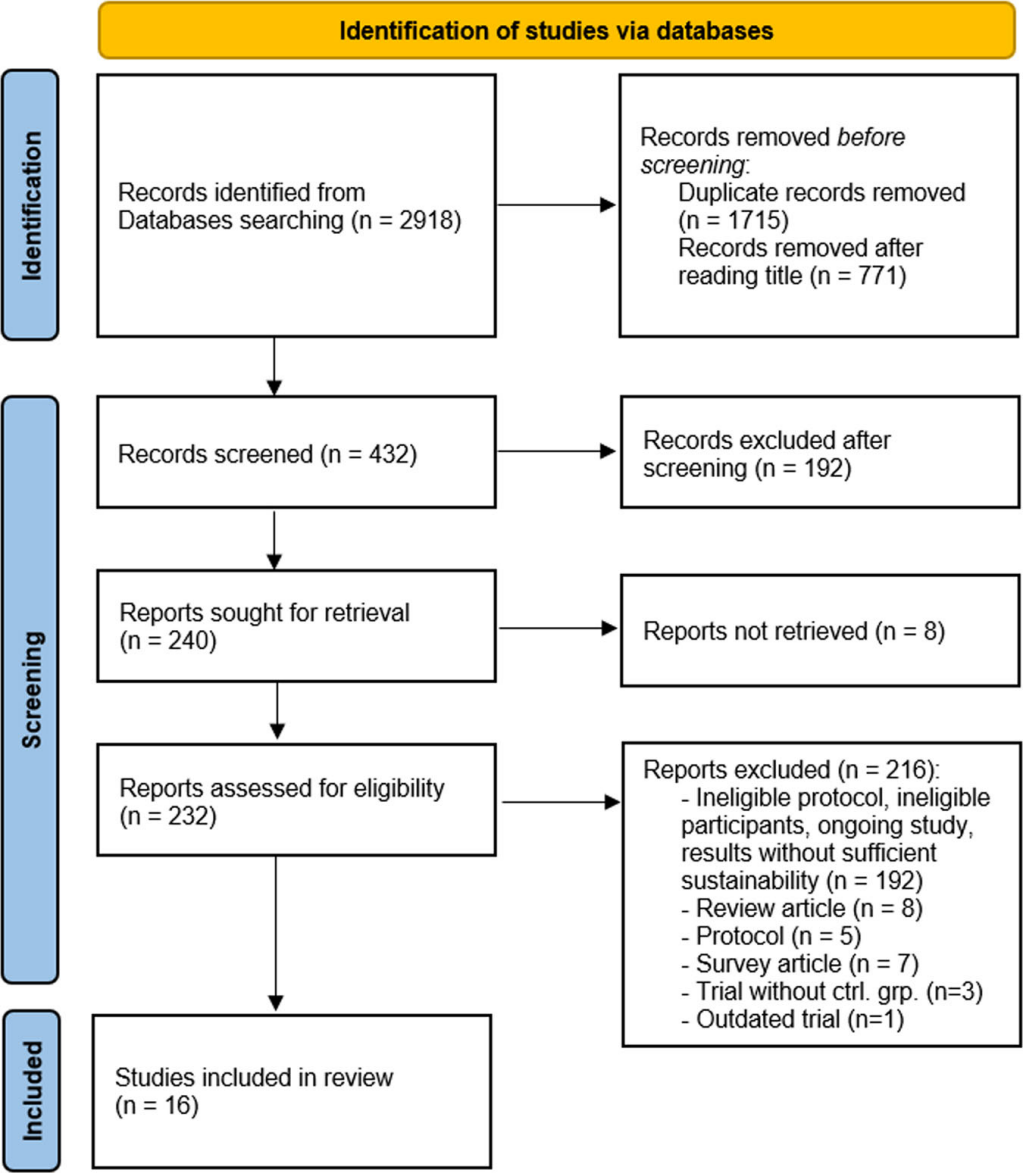
Flowchart and selection of studies to conduct the review based on the PRISMA guidelines. Workflow diagram describing the process for selecting articles aligned with the systematic review, detailing the studies from identification, screening, and final selection.

Following the removal of duplicate articles and those with non-matching titles, 432 records were retrieved for screening. The selection criteria identified a total of 16 studies (see Fig. 1). The main and supplementary outcomes are usually presented as abbreviations; therefore, Table 2, which contains the abbreviations and acronyms used in this systematic review, has been included. Details of the chosen studies, also referred to as articles, were provided in Tables 3 and 4, numbered chronologically based on their publication dates.

**Table 2:** Abbreviations and acronyms used.

Abbrev.	Meaning
COPD	Chronic Obstructive Pulmonary Disease
PR	Pulmonary rehabilitation
WBPTR	Web-based pulmonary telerehabilitation
EP	Exercise performance
HRQL	Health-Related Quality of Life
6MWT	Six minute walking test
SPD	Steps per day
CRQ-SAS	Chronic Respiratory Disease Questionnaire Self-Administered Standardised
SGRQ	St. George’s Respiratory Questionnaire
CAT	COPD Assessment Test
CCQ	Clinical COPD Questionnaire score
CRQ-D	Chronic Respiratory Disease Questionnaire - dyspnoea
mMRC	Modified Medical Research Council dyspnoea Scale
BCKQ	Bristol COPD Knowledge Questionnaire
BODE	Body Mass Index, Airway Obstruction, dyspnoea and Exercise Tolerance
MET	Metabolic equivalent
MFI	Multidimensional Fatigue Inventory
HADS	Hospital Anxiety and Depression Scale
ESWT	Endurance shuttle walk test
FEV_1_ %	Forced Expiratory Volume in one second, predicted value

**Table 3:** Set of studies reporting WBPTR, detailing the characteristics of the participants and the programme.

N.	Author	Total users	WB users	Patient ages: I/C	FEV1 % pred.: I/C	Oxygen Users %: I/C	Current Smok. %: I/C	Training kind	Freq.	Equipment	Programme
1	Nguyen, 2013^22^	84	43	68.5 ± 11 / 68.2 ± 9.9	53.3 ± 20.4 / 50.6 ± 18.2	26 / 24	5 / 5	Combin.	3	Treadmill, dumbbells	Endurance and arm strengthening exercise through routines displayed on the web.
2	Tabak, 2013^32^	34	16	65.2 ± 9 / 67.9 ± 5.7	48.7 ± 16.7 / 56.4 ± 10.6	NR	7.1 / 18.8	Extensive	4	None	Execute activities during the day to approach the reference shown.
3	Moy, 2015^33^	238	154	67 ± 8.6 / 66.4 ± 9.2	NR	22.7 / 25	26.6 / 21	Extensive	7	None	Walking programme based on monitoring a pedometer through the website.
4	Voncken, 2015^37^	1307	658	57.7 ± 7.3 / 57.6 ± 7.2	NR	NR	36.6 / 31.7	Combin.	5	None	Web-based self-management application based on physical activity and smoking habit monitoring with personalized feedback.
5	Moy, 2016^34^	238	154	67 ± 8.6 / 66.4 ± 9.2	NR	22.7 / 25	26.6 / 21	Extensive	5	None	Long-term home exercises were accessed online, offering personalized instructions and forum access.
6	Vorrink, 2016^23^	157	85	62 ± 9 / 63 ± 8	59 ± 20 / 53 ± 15	NR	NR	Extensive	5	None	A mobile app establishes objectives and offers routines along with positive feedback to inspire physical activity.
7	Wan, 2017^24^	109	57	68.4 ± 8.7 / 68.8 ± 8.7	60.2 ± 21.2 / 65.2 ± 21.9	26.3 / 21.2	36.1 / 34.6	Extensive	7	None	Personalized home routines via a web platform, tailored to the weather conditions.
8	Bourne, 2017^25^	90	64	69.1 ± 7.9 / 71.4 ± 8.6	58 ± 23.6 / 60.5 ± 20.1	NR	14 / 23	Combin.	5	Weighted bars, barbells	Ten online physiotherapist-designed exercises and educational videos about the disease and therapy.
9	Chaplin, 2017^36^	103	51	66.4 ± 10.1 / 66.1 ± 8.1	58.7 ± 29.1 / 55 ± 20.5	NR	NR	Combin.	2	Hand weights, dumbbells	Web platform based programme to access upper and lower extremity training with dumbbells.
10	Kessler, 2018^26^	319	157	67.3 ± 8.9 / 66.6 ± 9.6	37.8 ± 12.4 / 36.4 ± 12.3	NR	21.7 / 21	Extensive	NR	None	Self-management programme with home telemonitoring, care coordination, and medical management accessible online.
11	Robinson, 2019^27^	112	59	68.7 ± 8.9 / 68.9 ± 7.9	60.0 ± 20.9 / 65.0 ± 21.8	NR	NR	Extensive	3	None	Online platform aimed at providing objectives, feedback, and motivational educational content with social support.
12	Galdiz, 2020^28^	81	41	62.3 ± 8.2 / 63 ± 6.6	42.1 ± 14.6 / 45.9 ± 17.2	NR	NR	Combin.	3	Weight lifting, bike	Home cycling and resistance exercise.
13	Jiménez, 2020^29^	36	19	68.1 ± 6.6 / 68.1 ± 7	45 ± 15.3 / 43.1 ± 13.6	52.9 / 52.6	17.6 / 26.3	Extensive	7	None	HappyAir web-based programme and mobile app for maintaining physical activity.
14	Wan, 2020^35^	109	57	68.4 ± 8.7 / 68.7 ± 7.9	60.2 ± 21.1 / 65.2 ± 21.9	NR	38.6 / 34.6	Extensive	7	None	Website-based programme with feedback, goal setting, COPD education, and community forum.
15	Robinson, 2021^30^	153	75	69.2 ± 7.2 / 70.4 ± 7.3	60.6 ± 23.1 / 61.5 ± 19.8	25 / 28	NR	Extensive	NR	None	Web-based self-care programme with feedback on weekly goals and objectives. Incorporates a forum for disease education.
16	Tabak, 2022^31^	12	12	64.1 ± 9 / 62.8 ± 7.4	50 ± 20.9 / 36 ± 20.4	NR	33.3 / 33.3	Combin.	7	None	Telehealth platform with 4 modules: trainer, exercise programme, COPD self-management, and tele-consulting.

NR Not reported, Patient ages and FEV_1_ % predicted were presented as MD ± SD values for intervention/control groups.

**Table 4:** Set of studies reporting the content of web-based pulmonary telerehabilitation and the outcomes.

N.	Author	Supervision	Ed.	Wks.	Devices	Outcomes
1	Nguyen, 2013^22^	Collaborative self-monitoring of exercise and respiratory symptoms	Yes	12	Spirometer, Computer	CRQ-SAS, 6MWT, Arm lifts
2	Tabak, 2013^32^	Followed by a completed diary on the web portal	No	4	Pedometer, smartphone, Computer	Steps per day, CCQ, mMRC, MFI
3	Moy, 2015^33^	Online monitoring, weekly performance evaluation.	Yes	16	Pedometer Omron HJ-720 ITC, Computer	SGRQ, Steps per day
4	Voncken, 2015^37^	The use of the application was monitored by the research team and notices were sent by e-mail.	No	24	Computer	Smoking Cessation
5	Moy, 2016^34^	Monitoring through an online platform, personalised individual goals, iterative feedback.	Yes	48	Omron pedometer, Computer	SGRQ, Steps per day
6	Vorrink, 2016^23^	The therapist supervises patients individually through a web page accessing their results.	No	48	Accelerometer HTC, Smartphone, Computer	6MWT, CRQ-SAS, Steps per day, MET
7	Wan, 2017^24^	Personalised supervision through the web platform when reaching assigned goals	Yes	13	Omron Pedometer, Eaglet Spirometer, Computer	6MWT, SGRQ, mMRC, BCKQ
8	Bourne, 2017^25^	Online adjusting the intensity of 10 routines weekly based on their progress	Yes	7	Computer	6MWT, CAT, SGRQ, HADS, mMRC
9	Chaplin, 2017^36^	Weekly supervision by the physiotherapist via email or phone calls	Yes	8	Computer	CRQ-D, ESWT
10	Kessler, 2018^26^	Directly with the physician and in care coordination through an e-health online platform	Yes	48	NOWOX device, Computer	BODE, Exacerbations, 6MWT, HADS, Smoking habits, SGRQ
11	Robinson, 2019^27^	Through the web application	Yes	12	Omron Pedometer, Computer	6MWT, Steps per day
12	Galdiz, 2020^28^	Remote monitoring by physical therapist with feedback	Yes	48	Smartphone, Oximeter, Computer	6MWT, CRQ-SAS, BODE
13	Jiménez, 2020^29^	Therapeutic educators monitor HappyAir group progress and help patients with questions.	Yes	48	Smartphone, Computer	CAT, 6MWT, SGRQ
14	Wan, 2020^35^	Through the platform and every 3 months with phone calls for follow-up evaluations	Yes	60	Omron Pedometer, Computer	Steps per day
15	Robinson, 2021^30^	Web platform and phone calls	Yes	24	Computer, Fitbit Zip pedometer	6MWT, SGRQ, mMRC, BCKQ, DSC
16	Tabak, 2022^31^	Primary and secondary care professionals online supervise patients by monitoring their progress	Yes	36	Smartphone, Accelerometer, Computer	6MWT,CCQ, MFI

The total number of participants in these studies was 3190, with 53.2% (1697 participants) using WBPTR, while the remainder belonged to control groups. For the intervention group, participants had mean ages ranging from 57.7 to 69.2 years, with forced expiratory volume in one second (FEV_1_) ranging from 37.8% to 60.6% predicted. Meanwhile, for the control group, mean participant ages ranged from 57.6 to 70.4 years, with FEV_1_ ranging from 36% to 65.2% predicted (Table 3). Table 3 also includes the percentage of oxygen users and current smokers in both the intervention and control groups.

Among all studies, 81.3% reported the FEV_1_ % predicted (*n* = 13), 37.5% reported oxygen users (*n* = 6), 68.75% reported current smoking status (*n* = 11), and 93.75% reported gender (*n* = 15). Regarding the race of participants, two studies included percentages of various races (12.5%), four clarified the percentage of Caucasian participants (25%), and 62.5% did not include this information (*n* = 10).

The type of training varies across studies (Table 3: Training type). We categorise them into two main groups: Intensive training, which includes exercises and weight lifting in short sessions; extensive training, which involves activities like walking and cycling in longer sessions (*n* = 10, 62.5%); and a combination of both (*n* = 6, 37.5%). Session frequency was measured weekly (Table 3: Freq.), indicating the minimum number of days to be completed. 87.5% of studies reported this (*n* = 14). Regarding the duration lengths of the programmes (Table 4: Wks.), 18.75% (*n* = 3) of the articles specified trial lengths between 4 and 8 weeks, 37.5% (*n* = 6) between 12 and 24 weeks, and seven studies (43.75%) indicated a length longer than 24 weeks. Additionally, a large proportion of studies (75%) did not report the required equipment to perform the tasks (Table 3).

Web-based platforms enable digital reporting of participants’ progress. The supervision of goal achievement by therapists varies across studies, either using the web platform alone for feedback (*n* = 5, 31.25%) or enhancing it with telephone calls (*n* = 4, 25%), text messages (*n* = 2, 12.5%), or email (*n* = 4, 25%). Moreover, the web-based telerehabilitation feature allows for the inclusion of additional information accessible to users. Consequently, only 26.32% of articles lack any educational content regarding the disease. Conceivably, every study require a computer to access the web platform. However, access can also be done through devices such as tablets and smartphones (*n* = 4, 25%). Accessories such as spirometers (*n* = 2, 12.5%), pedometers (*n* = 7, 43.75%), oximeters (*n* = 1, 6.25%), and accelerometers (*n* = 2, 12.5%) were also used to collect physiological information.

All trials include at least some objective or subjective measurement of the progress in the patient’s recovery. We distinguish between two groups: those suitable for meta-analysis (primary outcomes) and those studied independently (secondary outcomes). For the meta-analysis, measurements from each article were extracted, normalising values when necessary (using SI units).

Primary outcomes were related to: a) Measurements of exercise performance (EP), where 62.5% of studies report the 6MWT (*n* = 10) and 37.5% study steps per day; b) Questionnaires to evaluate Health-Related Quality of Life (HRQL), where seven articles include the SGRQ (43.75%) and 18.75% report the CRQ-SAS (*n* = 3); c) Questionnaires about dyspnoea, where 25% of studies (*n* = 4) report the CRQ-D (independently or as part of CRQ-SAS), while three articles (18.75%) include the mMRC.

On the other hand, secondary outcomes consider: a) Arm lifts (*n* = 1, 6.25%) and the Endurance Shuttle Walk Test (*n* = 1, 6.25%), both related to EP; b) The CAT (*n* = 2, 12.5%) and the CCQ (*n* = 2, 12.5%), both related to HRQL; c) Measurements related to disease knowledge: the Bristol COPD Knowledge Questionnaire (*n* = 2, 12.5%); d) Fatigue-related health status: the Metabolic Equivalent (*n* = 1, 6.25%) and Multidimensional Fatigue Inventory (*n* = 2, 12.5%); e) Physical status of patients, the Body Mass Index, Airway Obstruction, Dyspnoea, and Exercise Tolerance (*n* = 2, 12.5%); and f) other measures such as the Hospital Anxiety and Depression Scale and COPD exacerbations, both included in at least one of the studies.

### Risk of bias in the included studies

**Fig. 2:**
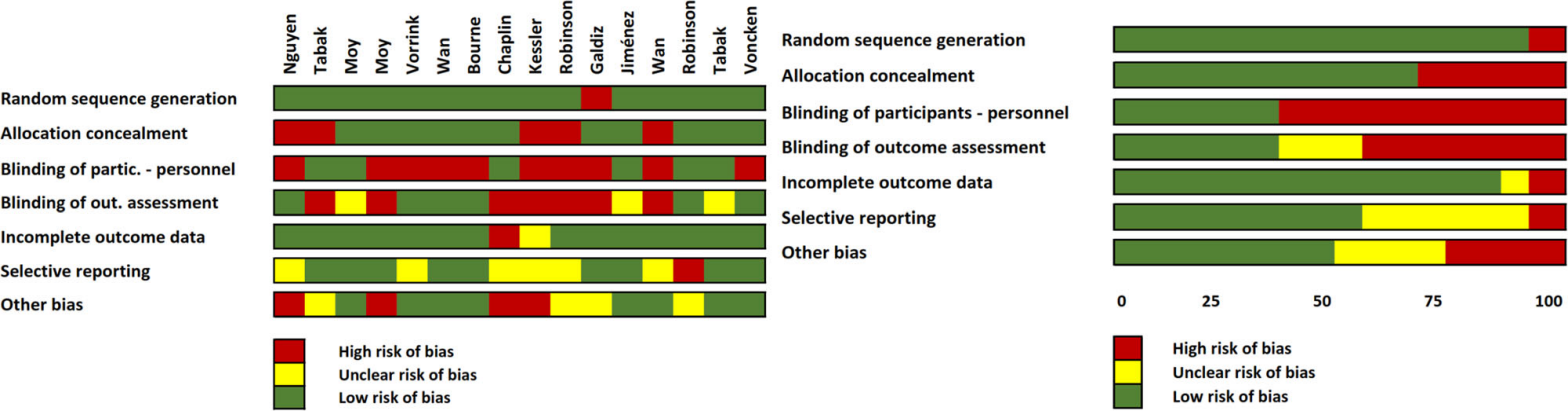
Risk of bias for the included studies. On the right, the total percentage for the Risk of Bias (ROB) is displayed, while on the left, each risk of bias item is detailed for each author.

The risk of bias in each of the selected studies was depicted in Fig. 2, indicating a high likelihood of bias (due to the study nature) in blinding of participants and personnel, and blinding of outcome assessment, with 62.5% of articles showing high or unclear ROB.

### Primary outcomes: meta-analysis

A total of thirteen studies provide quantifiable information covering Exercise Performance. The analysis of the 6WDT and the SPD was discussed in this subsection. Studies reporting quantifiable information were included in the meta-analysis and those that did not were analysed independently.

**Fig. 3:**
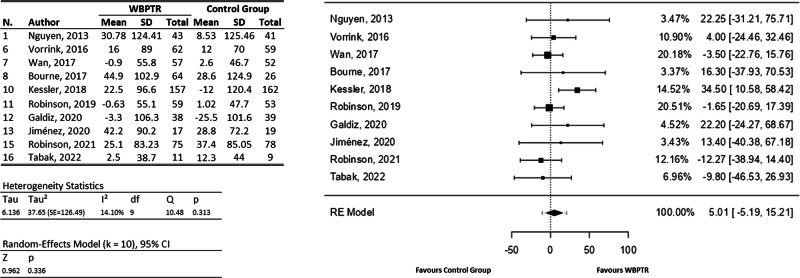
Six-minute walking test forest plot. On the left, a tabulation of the information extracted from each study is provided, covering both the WBPTR group (583 participants) and the control group (538 participants). On the right, a graphical representation shows the incidence of each study based on the number of patients.

With the information available from the 6WDT, ten studies (Fig. 3) involving a total of 1121 participants were included in the meta-analysis^22–31^. The observed mean differences ranged from −12.27 to 34.50, with more estimates favouring WBPTR (60%). The estimated average mean difference based on the random-effects model was $\hat{\mu }$ = 5.00 (95% CI: −5.19 to 15.21). Therefore, based on the values dimension, the average outcome did not differ significantly from zero (z = 0.96, *p* = 0.34). A 95% prediction interval for the true outcomes was given by −10.76 to 20.77. Hence, although the average outcome was estimated to favour WBPTR, in some studies the true outcome were in fact favouring traditional care^30,31^.

**Fig. 4:**
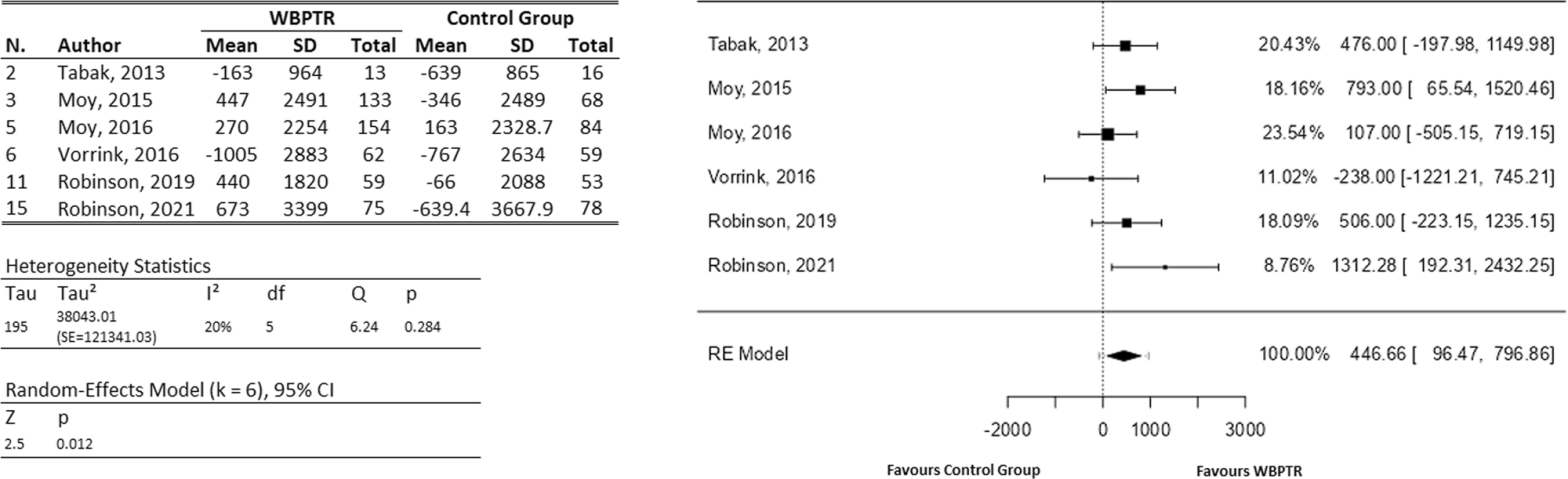
Steps per day forest plot. On the left, a tabulation of the information extracted from each study is provided, covering both the WBPTR group (496 participants) and the control group (358 participants). On the right, a graphical representation shows the incidence of each study based on the number of patients.

Regarding the SPD, six articles (involving a total of 854 participants)^23,27,30,32–34^ provide enough information for a meta-analysis (Fig. 4), whereas the study of Wan and colleagues^35^ was analysed independently. For the meta-analysis, the observed mean differences ranged from −238.00 to 1312.28, with the majority of estimates favouring web-based telerehabilitation approaches (83%). The estimated average mean difference based on the random-effects model was $\hat{\mu }$ = 446.66 (95% CI: 96.47 to 796.86). Therefore, the average outcome differed significantly from zero (z = 2.5, *p* = 0.01), denoting better results when using WBPTR. A 95% prediction interval for the true outcomes was given by −71.78 to 965.09. Hence, although the average outcome was estimated to be positive, one study favours traditional care^23^. On the other hand, the results obtained by Wan et al.^35^ suggest that no significant statistical differences were reached when comparing both traditional and WBPTR programmes.

Considering the Health-Related Quality of Life, ten studies include either the St. George’s Respiratory Questionnaire or the Chronic Respiratory Disease Questionnaire Self-Administered Standardised, both of which were analysed subsequently.

**Fig. 5:**
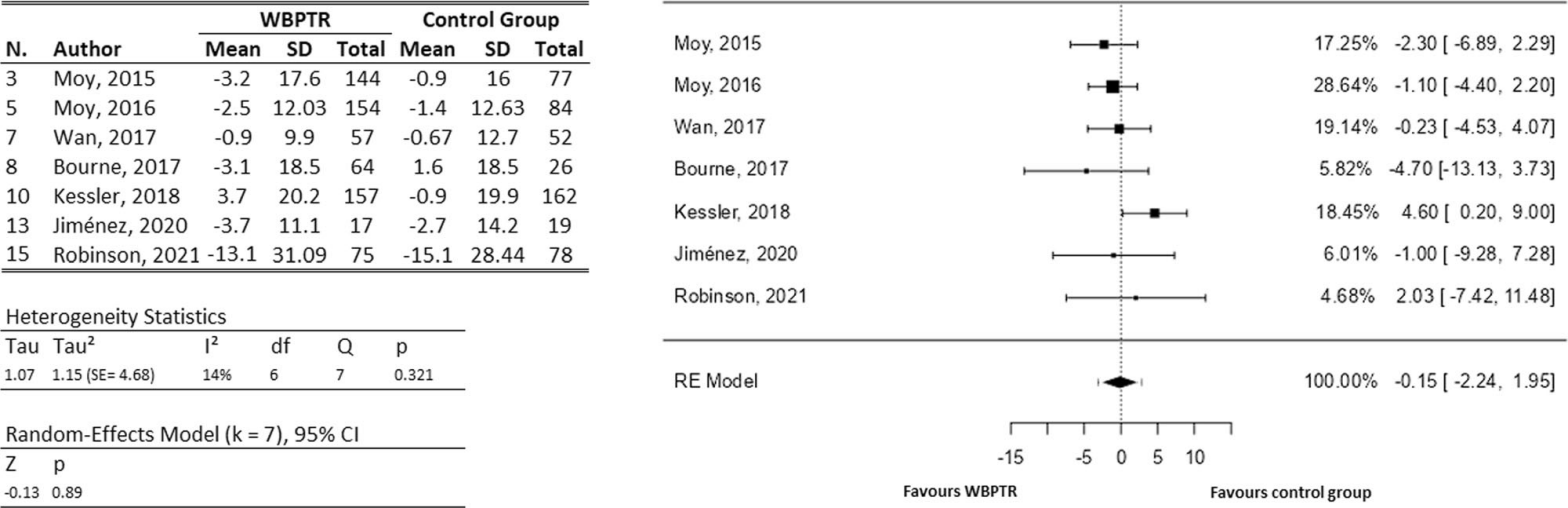
St. George’s Respiratory Questionnaire forest plot. On the left, a tabulation of the information extracted from each study is provided, covering both the WBPTR group (668 participants) and the control group (498 participants). On the right, a graphical representation shows the incidence of each study based on the number of patients.

For the SGRQ (Fig. 5), all articles mentioning its use provide sufficient information to be included in the meta-analysis. Therefore, a total of seven studies were considered^24,25,29,30,33,34,36^, involving a total of 1166 participants. The observed mean differences ranged from −4.70 to 4.60, with more of estimates favouring WBPTR (71%). The estimated average mean difference based on the random-effects model was $\hat{\mu }=-0.15$ (95% CI: −2.24 to 1.95). Therefore, the average outcome did not differ significantly from zero (z = −0.14, *p* = 0.89). A 95% prediction interval for the true outcomes was given by −3.12 to 2.83. Hence, although the average outcome was estimated to be negative, in two studies the true outcome was in fact positive (favours traditional care)^26,30^.

**Fig. 6:**
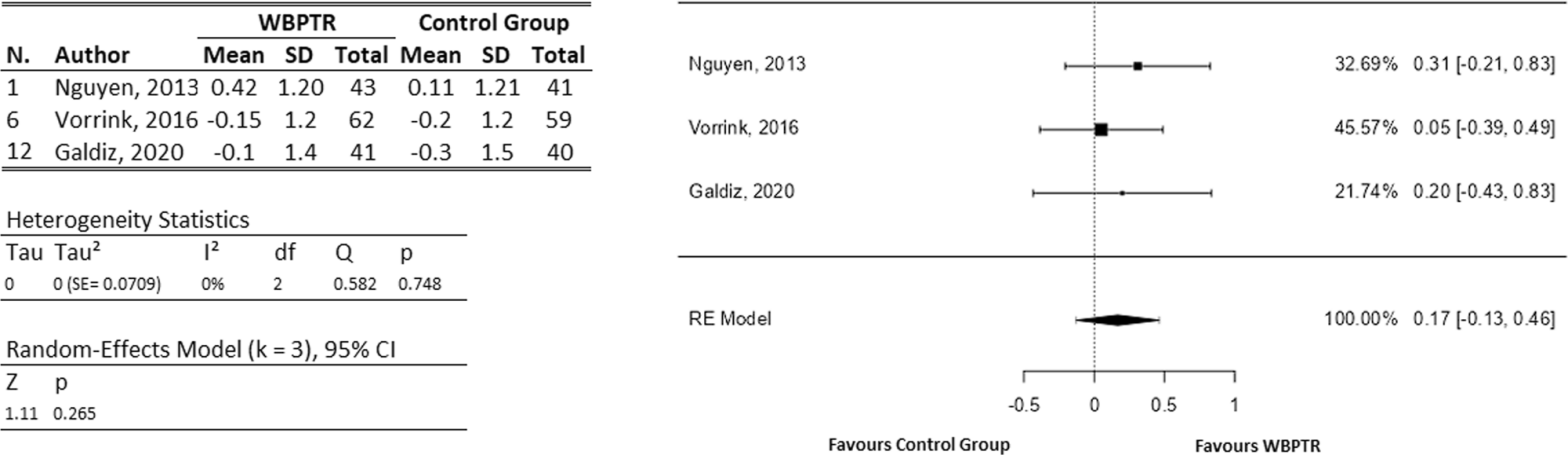
Chronic Respiratory Disease Questionnaire Self-Administered Standardised (CRQ-SAS) forest plot. On the left, a tabulation of the information extracted from each study is provided, covering both the WBPTR group (146 participants) and the control group (140 participants). On the right, a graphical representation shows the incidence of each study based on the number of patients.

In a case similar to the SGRQ, all trials that suggest measuring the CRQ-SAS provide sufficient information to analyse their results jointly (Fig. 6). Thus, data from three studies^22,23,28^ were included in the meta-analysis, involving 286 participants. The observed mean differences ranged from 0.05 to 0.31, with all the estimates favouring WBPTR. The estimated average mean difference based on the random-effects model was $\hat{\mu }=0.17$ (95% CI: −0.13 to 0.46). Therefore, the average outcome did not differ significantly from zero (z = 1.11, *p* = 0.27).

This subsection analyses the seven studies that include the Chronic Respiratory Disease Questionnaire and the Modified Medical Research Council scale, both of which refer to dyspnoea.

**Fig. 7:**
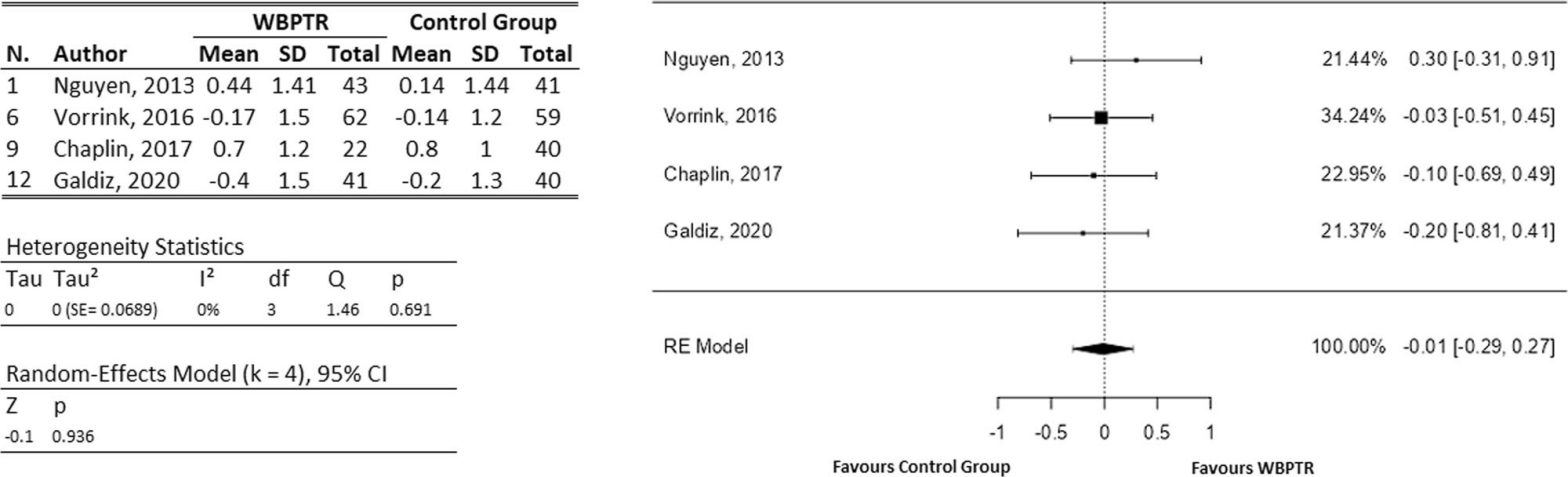
Chronic Respiratory Disease Questionnaire - dyspnoea (CRQ-D) forest plot. On the left, a tabulation of the information extracted from each study is provided, covering both the WBPTR group (168 participants) and the control group (180 participants). On the right, a graphical representation shows the incidence of each study based on the number of patients.

Either independently or as part of the CRQ-SAS, four studies considers the dyspnoea^22,23,28,36^, all including sufficient information to analyse them together in the meta-analysis (Fig. 7) and involving a total of 348 participants. The observed mean differences ranged from −0.20 to 0.30, with the majority of estimates being negative (75%), i.e., favouring traditional care. However, the average outcome was practically zero (z = −0.08, *p* = 0.94). The estimated average mean difference based on the random-effects model was $\hat{\mu }=-0.01$ (95% CI: −0.29 to 0.27).

**Fig. 8:**
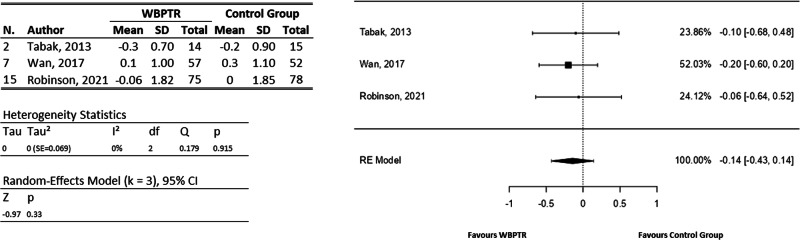
Modified Medical Research Council dyspnoea Scale forest plot. On the left, a tabulation of the information extracted from each study is provided, covering both the WBPTR group (146 participants) and the control group (145 participants). On the right, a graphical representation shows the incidence of each study based on the number of patients.

For the mMRC, three studies^24,30,32^ provide information to include them in the meta-analysis (Fig. 8), whereas the study of Bourne et al.^25^ was analysed independently. A total of 291 participants were involved. Thus, for the data analysed together, the observed mean differences ranged from −0.20 to −0.06, with all of them favouring WBPTR. The estimated average mean difference based on the RE model was $\hat{\mu }=-0.14$ (95% CI: − 0.43 to 0.14). Therefore, the average outcome differ from zero, but not significantly (z = −0.98, *p* = 0.33). Moreover, the independent results obtained from Bourne et al.^25^ show equivalent benefits between WBPTR and traditional care, suggesting an improvement in dyspnoea, although there were no significant differences between treatments.

#### Remark 1

According to Cook’s distances, the study by Kessler and colleagues^26^ could be considered overly influential for the 6WDT and the SGRQ. For the remaining outcomes, no study was considered to be overly influential.

#### Remark 2

According to the Q-test, there was no significant heterogeneity in the true outcomes for any of the studies.

#### Remark 3

Examination of the studentised residuals revealed that none of the meta-analyses developed presented outliers. None of the studies had a value greater than ± 2.81, ± 2.64, ± 2.69, ± 2.39, ± 2.49, and ± 2.39 for 6MWT, SPD, CRQ-SAS, SGRQ, CRQ-D, and mMRC, respectively.

### Secondary outcomes

For exercise performance, arm lifts and the Endurance Shuttle Walk Test (ESWT) were also considered secondary outcomes. In this case, Nguyen and colleagues^22^ mention that at six and twelve months, WBPTR participants perform more arm lifts compared to the control group, but numerical data was not provided. On the other hand, the ESWT was analysed in the study by Chaplin et al.^36^, which shows a significant change at the end of treatment for both the control and intervention groups, but no significant difference between the groups.

Secondary outcomes for Health-Related Quality of Life include the COPD Assessment Test and the Clinical COPD Questionnaire. In the study by Bourne and colleagues^25^, the disparity in the CAT scores between groups favoured the WBPTR, with a difference of −1.0. Similarly, the mean evolution graph indicates a 3.6-point difference between the HappyAir group and the control group after 12 months of follow-up in the study by Jiménez et al.^29^. This suggests a better quality of life in the WBPTR group compared to the baseline evaluation, as a 2.5-point difference in the CAT questionnaire was considered clinically relevant. Moreover, two articles in the set of studies mention the CCQ^31,32^, both showing improvement within groups, although there were no significant changes between the treatment groups.

Although in a smaller set, several studies consider parameters such as COPD knowledge, fatigue, physical condition of the patients, anxiety, depression, among others. To encompass these subjective measurements, this section includes the results of these studies.

The Bristol COPD Knowledge Questionnaire (BCKQ) was measured and compared between the intervention group and the control group in studies by Wan et al.^24^ and Robinson et al.^30^. Both articles show a statistically significant change compared to baseline; however, no noticeable difference was observed when comparing treatments.

The Multidimensional Fatigue Inventory (MFI) was analysed in two studies^31,32^, examining all sub-items: general fatigue, physical fatigue, reduced activity, reduced motivation, and mental fatigue. Similar to other studies mentioned earlier, both studies show improvement in general fatigue from the end of treatment compared to the beginning. However, no significant difference was observed between the WBPTR group and the control group.

Additionally, the BODE (Body Mass Index, Airway Obstruction, Dyspnoea, and Exercise Tolerance) index was analysed in Kessler et al.^26^ and Galdiz et al.^28^. The results from these two trials differ significantly in both within-group and between-group comparisons. Galdiz and colleagues^28^ report non-significant differences between groups, while Kessler and colleagues^26^ indicate notable differences both within groups and between groups, showing superior results with WBPTR compared to traditional care and usual care methods.

Additionally, the Metabolic Equivalent of Task (MET) was analysed solely in the study by Vorrink et al.^23^, which indicates no statistically significant difference over the entire treatment duration, both within groups and in the comparison between groups.

Regarding smoking habits, Voncken et al.^37^ did not find significant differences in any comparison, whereas Kessler and colleagues^26^ show that more patients in the WBPTR group stopped smoking during the study.

When analysing parameters related to intervention from health centres and hospitals, several measures provide information on treatment comparisons. Specifically, the Hospital Anxiety and Depression Scale (HADS) was analysed by two studies^25,26^, with only Bourne and colleagues^25^ reporting a significant reduction favouring WBPTR. Lastly, COPD exacerbations were examined in one study^26^, noting a reduction for WBPTR that may potentially be influenced by external factors unrelated to the treatment.

## Discussion

This review outlines the key aspects of web-based pulmonary rehabilitation platforms, detailing the characteristics of these programmes, types of exercises and education, participant health status and demographics, duration of rehabilitation, devices and equipment used, and measured outcomes. This information will provide clinicians with the necessary tools when delivering web-based telerehabilitation.

Various differences were reported among the studies, such as how rehabilitation progress was tracked, the types of devices and equipment used, the nature of the outcomes, the method of delivering education, the type of training, and the frequency of sessions. These variations complicate the design of an optimal programme for delivering web-based telerehabilitation. Additionally, the assumption that participants have access to a computer or smartphone and the internet, as well as necessary equipment (such as treadmills, dumbbells, or exercise bikes), can create a barrier for those from lower socioeconomic backgrounds. Regarding web platforms, 75% of the studies did not report the specific web portal used, nor clarify whether it was purpose-built, the security features included, or if there was a cost associated. Only four studies^25,29,36,37^ mention the platform used, without indicating whether there was a cost involved or if it was covered for the participants. Clarifying the costs behind the management of these web tools, their maintenance, and data processing would provide clear information for choosing one, allowing for an understanding of their advantages and limitations.

An emergency action plan was also not included in 68.75% of the studies (*n* = 11), despite its importance when working with individuals with moderate to severe COPD, with ages reaching an average of 69.1 years (for the intervention group). In contrast, the works of Nguyen et al.^22^, Kessler et al.^26^, and Galdiz et al.^28^ provide detailed support to participants, even incorporating emergency states detectable by the web platforms. Two additional studies report the steps to be taken in case of emergencies, but it was unclear whether these were part of the platform or merely guidelines on how to act if an adverse event occurs^30,34^.

Although 93.75% of WBPTR programmes report the gender of participants, 62.5% of the programmes omit reporting the race and ethnicity of users (*n* = 10). Of the remaining 37.5%, four studies^22,24,30,35^ indicate the Caucasian percentage, and only two^33,34^ provide a detailed percentage breakdown of the participants. Given the structure and requirements of WBPTR programmes, reporting race and ethnicity could highlight disparities among participants, enabling the adaptation of treatments to meet the specific needs of each user group.

When dealing with predominantly elderly patients, their comfort with technology or at least their educational level should be documented. However, 68.75% (*n* = 11) of the studies did not include such reports. Only Nguyen et al.^22^ and colleagues document users’ comfort with internet use, while four studies^27,30,32,37^ report only the the participants’s education level. Not knowing the relationship between participants and their proficiency with technology increases the difficulty in detecting data entry errors and accessing the web platform’s resources.

Through meta-analysis, it was shown that WBPTR programmes demonstrate a non-significant improvement compared to traditional care in primary and secondary outcomes. The meta-analysis of measurements obtained for any of the outcomes did not exceed the minimum clinically important difference, but it was noted that the mean standardized mean difference reaches 75% of the proposed MCID. Furthermore, the CAT (analysed independently) shows a notable difference in favour of WBPTR; however, these conclusions were not drawn from a meta-analysis. For all measurements included in the meta-analysis, low heterogeneity was observed, with the highest for the count of steps per day (I^2^ = 19.88%).

The findings from this review, although not significant, were in agreement with the improvements reported by Cox et al.^38^ and Michaelchuk^39^ when comparing 6MWT, SPD, CRQ-D or mMRC. However, contrary to the findings of Michaelchuk et al.^39^, which show improvements exceeding the MCID in SGRQ, our review found no significant difference. The type of supervision, quality of reporting by participants, or barriers related to technology use may explain these differences.

On the other hand, the percentage of education provided in the studies was noteworthy. Indeed, 81.25% of the studies include education for participants, either in a detailed manner through videos, online sessions, demonstrations, or interactive formats (*n* = 5, 31.25%), or in a secondary, unspecified manner. Additionally, emotional support and motivation were commonly integrated into WBPTR programmes (*n* = 12, 75%). However, parameters such as COPD knowledge did not show the expected significant improvement compared to the control group. Presumably, this was related to the participants themselves, who often encounter difficulties with web platforms, lack interest in learning, or were not adequately suited to the methods used within their age range.

This work highlighted common features used by web-based platforms for delivering rehabilitation, noting weaknesses that could be addressed in designing future trials. When working with older adults, it is recommended to have an emergency plan to detect adverse effects, leveraging the technology used in these studies to facilitate such alerts and increase users’ acceptance of these tools. Additionally, reporting participants’ compliance with internet usage (or alternatively, their level of education) is considered necessary to detect potential biases in progress reporting during recovery. This approach also allows for tailoring how information is delivered to effectively educate users, identifying strengths and weaknesses in each case. Finally, a complete report on the types of training, duration, equipment, and devices used can indicate the most suitable guidelines and which best fit the socioeconomic conditions of each group of participants.

Based on our findings, this review indicates that WBPTR did not show significant differences compared to traditional care. Although no significant improvements were observed in all measurements assessed, a slight improvement in exercise performance was found with WBPTR. However, there were no notable improvements in dyspnoea measurements or quality of life. Overall, WBPTR can be considered a complementary and accessible option to enhance the care and follow-up of patients with chronic obstructive pulmonary disease. Nevertheless, further studies and innovative developments in this field are necessary to optimise its effectiveness and ensure its applicability across various clinical scenarios.

The strengths of this review lie in its comprehensive coverage of information, encompassing four different objective measures (6MWT, steps per day, arm lifts, and ESWT), as well as thirteen measures based on scales and questionnaires widely used in the literature. However, due to the nature of the included articles, many were unable to blind participants (either medical staff or patients) in comparison to their control groups, or blind the type of outcome assessments. These limitations could potentially be addressed in future trials with improved trial protocols. Moreover, given the variety of articles proposing alternative rehabilitation strategies divergent from traditional care, there remains a possibility of excluding research that could make significant contributions. To mitigate this, meta-analyses have been structured to be reproducible and inclusive of future studies. Additionally, we acknowledge that the trials encompass patients with diverse conditions, and despite conducting heterogeneity analyses, the conclusions drawn may necessitate further study. Lastly, the requirement for various tools (such as a computer with internet access at home, basic web page navigation skills, and proficiency in smartphone applications) could introduce a high risk of bias in published and analysed results. These characteristics should be carefully considered before translating such studies into real-world applications.

## Data Availability

The data that support the findings of this study are available from the corresponding author upon reasonable request.
